# Association of transportation noise with cardiovascular diseases

**DOI:** 10.1002/clc.24275

**Published:** 2024-05-06

**Authors:** Badar ud Din Shah, Rohan Raj, Parvinder Kaur, Ali Karim, Raveena Bai Bansari, Amin Mehmoodi, Jahanzeb Malik

**Affiliations:** ^1^ Department of Cardiovascular Medicine Cardiovascular Analytics Group Islamabad Pakistan; ^2^ Department of Medicine Nalanda Medical College and Hospital Patna India; ^3^ Department of Medicine Crimean State Medical University Simferopol Ukraine; ^4^ Department of Medicine Ibn e Seena Hospital Kabul Afghanistan

**Keywords:** cardiovascular diseases, environmental health, noise mitigation, noise pollution, urban planning

## Abstract

This comprehensive article delves into the intricate and multifaceted issue of noise pollution, shedding light on its diverse sources, profound health implications, and the economic burden it imposes on societies. Noise pollution is an increasingly prevalent environmental challenge, impacting millions of people worldwide, often without their full awareness of its adverse effects. Drawing from a wealth of scientific research, the article underscores the well‐established links between noise pollution and a spectrum of health issues, including cardiovascular diseases, sleep disturbances, and psychological stress. While exploring the sources and consequences of noise pollution, the article highlights the urgent need for a holistic and collaborative approach to mitigate its impact. This entails a combination of regulatory measures, technological innovations, urban planning strategies, and public education campaigns. It is increasingly evident that the detrimental effects of noise pollution extend beyond physical health, encompassing mental and social well‐being. The article also addresses the synergistic relationship between noise pollution and other environmental stressors, emphasizing the importance of considering noise in conjunction with factors like air pollution and access to green spaces. It examines the potential of green spaces to mitigate the effects of noise pollution and enhance overall health.

## 1 INTRODUCTION

Air pollution is a well‐studied environmental stressor with known adverse health effects, particularly on cardiovascular disease (CVD). These effects include conditions such as acute myocardial infarction (MI), heart failure, arrhythmia, hypertension, and stroke.^1^ Particulate matter with a diameter of 2.5 μm (PM2.5) has been linked to approximately 8.8 million annual deaths worldwide, with CVD, including ischemic heart disease (40%) and stroke (8%), being major contributors to this mortality.^2^ Interestingly, while air pollution's impact on health is extensively researched, environmental noise, another common urban stressor, receives relatively less attention in scientific studies. This is despite the frequent coexistence of air pollution and noise in urban areas.^3^ In Europe, for example, a significant portion of the population is exposed to high levels of traffic noise, and this number is expected to rise due to urban growth and increased mobility demands.^3^ A key indicator of noise exposure is *L*_den_, which accounts for noise levels over a whole day, with penalties for nighttime and evening noise levels. Millions of people in Europe are exposed to high levels of traffic, railway, and aircraft noise, with projections indicating an increase in exposure.^3^ There is a clear projection that the number of people exposed (e.g., to road noise >55 dB[A]) will increase by 7.8% and to railway noise by 11.8% inside urban areas and by 16.4% and 8.7%, respectively, outside urban areas until the year 2030.^3^ In contrast, the number of people exposed to industrial noise will be strikingly reduced by almost 40% inside urban areas, whereas the number of people exposed to aircraft noise inside or outside metropolitan areas will remain unchanged.^3^ Annoyance due to noise and sleep disturbance caused by noise are also prevalent issues, with severe sleep disturbance being a significant risk factor for future cardiovascular events.^4^ Environmental noise from various sources is estimated to contribute to thousands of new cases of ischemic heart disease and premature deaths annually.^5^ The exposure to environmental noise from road traffic, railways, aircraft, and industry is estimated to contribute every year to approximately 48 000 new cases of ischemic heart disease and 12 000 premature deaths. Despite this significant health impact, guidelines for CVD prevention from organizations like the American Heart Association/American College of Cardiology, the Global Burden of Disease publications, and the European Society of Cardiology inadequately address transportation noise as a risk factor.^6^, ^7^ In this review, the focus is on the indirect cardiovascular health effects of transportation noise, beyond its auditory impact.^8^ The discussion covers the epidemiology of noise‐related CVD, and the pathophysiology of how noise affects vascular and cerebral function, including its impact on endothelial function, inflammation, oxidative stress, and metabolic disease. The review also explores the interaction between traditional cardiovascular risk factors like diabetes, hypertension, hypercholesterolemia, and smoking, and the emerging risk factor of environmental noise. Importantly, noise is shown to share many signaling pathways with these classical risk factors. In addition, the review delves into the potential for effective mitigation measures to reduce the cardiovascular side effects of noise exposure. Overall, it underscores the need for greater recognition and inclusion of transportation noise as a significant risk factor in CVD prevention guidelines and research efforts.

## 2 MECHANISM OF NOISE CAUSING CVD

**Figure 1:**
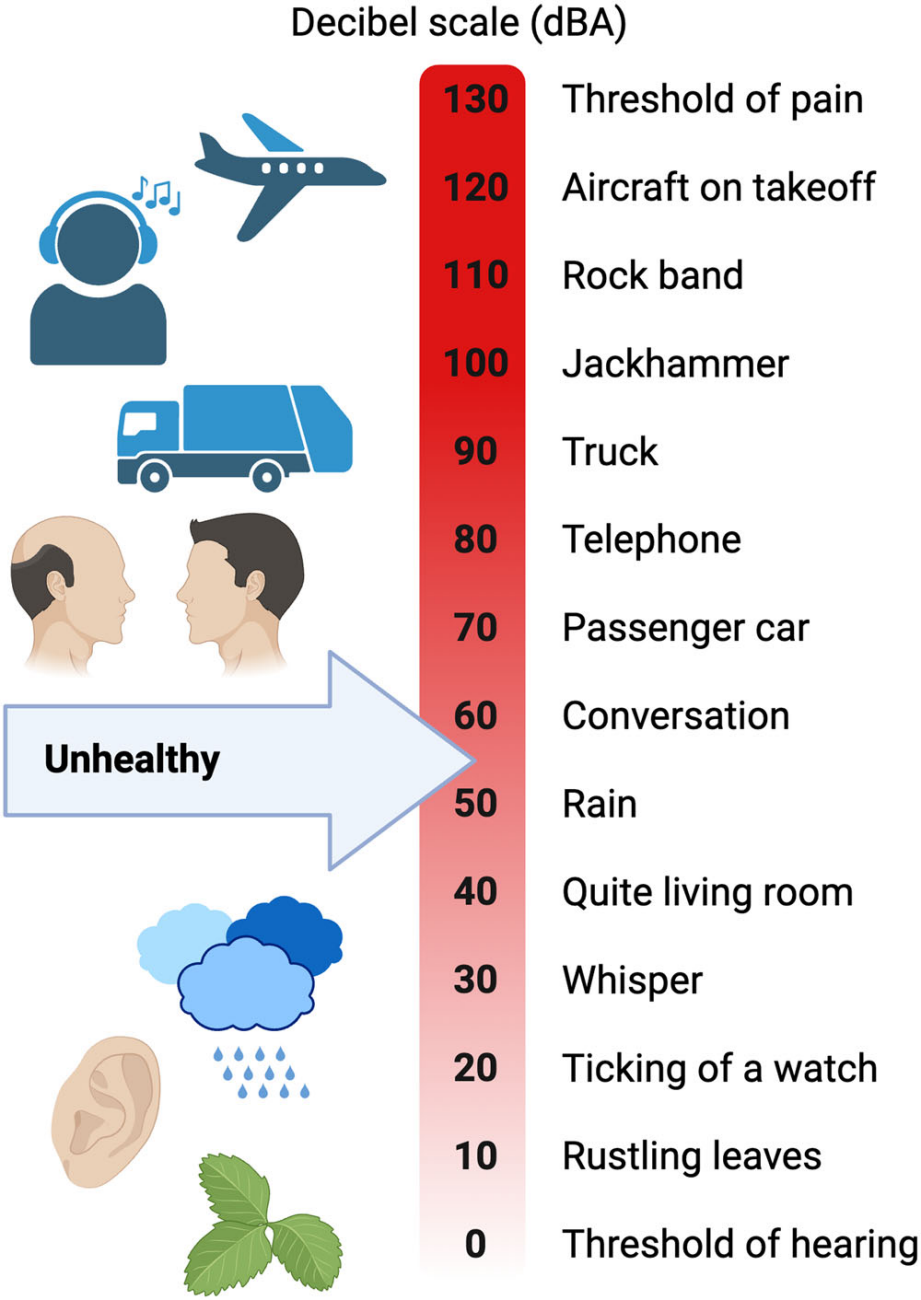
Stress signaling by noise.

According to Wolfgang Babisch's stress noise concept, there are two primary pathways by which noise exposure affects our health.^9^ These pathways are known as the “direct pathway” and the “indirect pathway.” The direct pathway involves exposure to very high decibel levels, typically exceeding 100 dB(A). In this case, noise at such extreme levels can directly damage the delicate structures of the ear, leading to hearing impairment or damage to the auditory organs. The indirect pathway, on the other hand, comes into play when noise levels are lower, typically falling in the range of 50–60 dB(A). In this scenario, noise disrupts various activities, including sleep and communication. This disturbance can trigger emotional stress responses in individuals, such as annoyance or even anger. These emotional responses are associated with physiological changes in the body, including an increase in cortisone levels (a stress hormone) and activation of the sympathetic nervous system (the “fight or flight” response). Over time, chronic stress responses resulting from noise exposure can contribute to the development of various cardiovascular risk factors.^8^ These risk factors include hypertension, elevated glucose and cholesterol levels, increased blood viscosity, and activation of the coagulation system. These risk factors are known contributors to CVD. In the long term, persistent stress responses to noise can lead to the development of CVD, including conditions like manifest hypertension, atherosclerosis, acute and chronic coronary syndromes, stroke, heart failure, and arrhythmia. Notably, acute exposure to nighttime noise‐related stress can even trigger a specific condition known as “stress cardiomyopathy” or “Takotsubo syndrome.” This condition is often referred to as “broken heart disease” because it can mimic the symptoms of a heart attack and is associated with extreme emotional stress.^10^ Figure 1 shows the stress signaling by noise and leading to CVDs.

## 3 CARDIOVASCULAR RISK FACTORS AND NOISE POLLUTION

### 3.1 Noise and sleep disturbance

There is a growing body of research highlighting the critical importance of getting an adequate amount of sleep for overall health. Insufficient sleep has been linked to a range of adverse health outcomes, including an increased prevalence of cardiovascular risk factors and a higher risk of cardiovascular events.^11^ Some of the cardiovascular risk factors associated with inadequate sleep include obesity, diabetes mellitus, and hypertension. These conditions are known contributors to heart disease and other cardiovascular problems. Polysomnographic investigations, which involve monitoring various physiological parameters during sleep, have provided insights into the side effects of acute noise exposure on sleep. These studies have shown that nighttime noise can significantly increase the likelihood of awakening reactions during sleep.^12^ This means that noise disruptions, even if they do not fully wake you up, can fragment your sleep and disrupt its quality. Recent prospective cohort studies have delved further into the relationship between road noise and sleep. For example, one study analyzed data on the use of sleeping medication and found a weak association with nighttime noise levels exceeding 55 dB(A).^13^ This suggests that people are exposed to high noise levels to seek sleep aids, possibly due to sleep disturbances caused by noise. Another study explored the connection between extreme noise annoyance and self‐reported sleep disturbance. It discovered an association between individuals who reported high levels of noise annoyance and sleep disturbances when followed up over time. This further underscores the disruptive impact of noise on sleep quality.^14^ However, despite these findings, it is worth noting that the quality of evidence in this area has only seen slight improvements since the World Health Organization (WHO) report and noise guidelines for the European region were published. There is still a need for more comprehensive studies that use objective measures or standardized subjective indicators of sleep quality to better understand the relationship between noise exposure and sleep disruption.

### 3.2 Noise and hypertension

The impact of noise on mental stress reactions has led to extensive research into its effects on blood pressure. Interestingly, the WHO conducted a comprehensive analysis that included over 35 cross‐sectional studies. Their findings indicated a modestly increased risk of prevalent hypertension (high blood pressure) associated with road traffic noise, with a risk ratio of 1.05 (95% confidence interval [CI], 1.02–1.08).^15^ In other words, the risk of developing hypertension was slightly elevated in individuals exposed to road traffic noise. However, subsequent studies have produced mixed results. Some investigations supported the association between traffic noise exposure and hypertension, while others did not. This inconsistency has led the WHO's expert panel to classify the evidence supporting a link between traffic noise and hypertension as “very low.”^16^ This classification suggests that the strength of the association is uncertain and that more research is needed to establish a clear and reliable estimate of the effect. It is worth noting that the association between noise and hypertension appears to be more pronounced during nighttime exposures compared to daytime exposures. The Hypertension and Exposure to Noise Near Airports study, for instance, found a significant link between nighttime aircraft noise and prevalent hypertension. In contrast, no such association was observed for daytime aircraft noise.^17^ This aligns with the understanding that nighttime noise events can have a more pronounced impact on physiological factors like arterial stiffness, endothelial function, and blood pressure, especially in individuals with established coronary artery disease.^18^ Since the publication of the WHO reports, which highlighted inconsistencies in the effects of noise on blood pressure, drawing reliable conclusions has become challenging. This uncertainty underscores the need for more high‐quality studies to better understand the relationship between noise exposure and its impact on blood pressure. Further research in this area can help inform public health measures and policies aimed at mitigating the adverse health effects of noise pollution, particularly in relation to hypertension and cardiovascular health.

### 3.3 Noise pollution and diabetes mellitus

Transportation noise, particularly from road and railway sources, has been suggested to be associated with diabetes mellitus type 2 (T2DM), a metabolic disease characterized by insulin resistance and impaired glucose regulation.^19^ The connection between noise and diabetes is multifaceted and involves several physiological and behavioral pathways. First, noise‐induced psychological stress disturbs the hypothalamic–pituitary–adrenal axis, leading to an increased release of cortisol, a stress hormone.^20^ Elevated cortisol levels can initiate metabolic changes that contribute to the development of diabetes. Noise also disrupts sleep patterns, leading to both too‐short sleep intervals and frequent interruptions of sleep. This sleep disturbance can result in several adverse effects, including low‐grade inflammation, reduced insulin sensitivity, impaired glucose regulation, and potentially dysregulation of appetite‐regulating hormones.^21^ These factors collectively contribute to the development of T2DM. Furthermore, noise annoyance itself can represent a behavioral pathway through which noise exposure may induce cardiometabolic diseases, including diabetes.^5^ Chronic annoyance caused by noise can contribute to chronic stress, which is linked to the development of metabolic disorders. Noise pollution is also associated with risk factors for diabetes, such as obesity and physical inactivity. These factors can increase the likelihood of developing T2DM in individuals exposed to noisy environments. While the WHO initially rated the evidence for the risk of developing T2DM due to noise exposure as very low to moderate in 2018, subsequent prospective studies have provided more compelling evidence.^15^ Specifically, the association between road noise exposure and T2DM remained significant even after adjusting for air pollution levels. Recent research extended this evidence by showing that long‐term exposure to road, railway, and potentially aircraft noise was associated with an increased risk of T2DM in a nationwide cohort of Danish adults, even after accounting for ambient air pollution levels.^22^ Importantly, combined exposure to noise from multiple sources, such as road and railway noise, appears to be particularly harmful. In a systematic review and meta‐analysis, a 5 dB(A) increase in noise exposure was associated with a 6% increase in the risk of diabetes, with T2DM primarily linked to air and road traffic noise.^23^

### 3.4 Annoyance and noise pollution

Noise annoyance, which stems from the disruptions caused by noise, is itself a result of stress responses triggered by noise pollution. Noise can disturb sleep, hinder communication, and disrupt various activities, leading to emotional reactions characterized by increased levels of circulating cortisone and activation of the sympathetic nervous system, which is responsible for the “fight or flight” response.^24^ Noise annoyance has been linked to various health issues, highlighting its role as a significant stressor. For example, studies have reported associations between noise annoyance and atrial fibrillation.^25^ It has also been shown to predict symptoms of depression, anxiety, and sleep disturbances. Interestingly, the impact of noise annoyance on health appears to vary by sex, with different risks associated with different genders. A novel neurobiological mechanism has recently been proposed to link transportation noise with CVD. Researchers found that in a group of individuals without pre‐existing CVD, the amygdala activity, a part of the brain's limbic system responsible for emotional responses, was quantified in response to road and aircraft noise.^26^ They used 18F‐fluorodeoxyglucose positron emission tomography‐computed tomography scans to investigate vascular inflammation (specifically in the aorta) within a 5‐year follow‐up period. In subjects with high noise exposure, there was evidence of vascular inflammation within the follow‐up period. In contrast, individuals with low noise exposure showed regular amygdala activity and no signs of vascular inflammation.^26^ Furthermore, those exposed to high noise levels within the 5‐year timeframe experienced a higher frequency of major adverse cardiovascular events. These events included CVD‐related deaths, MI, unstable angina, and cerebrovascular incidents. Mediation analysis revealed a sequence of events that explains how noise exposure can lead to cardiovascular problems: noise exposure → amygdala activation → activation of the sympathetic nervous system and the hypothalamic–pituitary–adrenal axis → systemic and vascular inflammation → atherosclerosis → increased risk of major adverse cardiovascular events. Importantly, it is worth noting that individuals with more resilience to noise pollution experience fewer cardiovascular side effects.^27^ This suggests that strategies to enhance an individual's ability to cope with noise‐related stress could be beneficial in reducing the negative health impacts of noise pollution.

### 3.5 Obesity and noise pollution

The evidence linking road traffic noise to obesity has significantly expanded since the previous evaluation by the WHO. At that time, only three cross‐sectional studies were available, leading to a conclusion of “very low‐quality evidence.”^28^, ^29^, ^30^ Since then, there has been notable progress in research in this area.^30^ Three longitudinal studies investigating the relationship between road traffic noise and measures of obesity in adults have been published. These studies have explored various.^28^, ^29^, ^30^ Although obesity, and although the specific measures may vary across studies, they collectively suggest a consistent pattern: road traffic noise exposure is associated with markers of adiposity and obesity. However, it is important to note that there is still a need for more research, particularly in understanding the effects of railway and aircraft noise on obesity. This is partly because previous studies in this regard have yielded inconsistent results, and a more comprehensive body of evidence is required to draw definitive conclusions about the impact of these specific noise sources on obesity. In summary, while the evidence connecting road traffic noise to obesity has strengthened in recent years, further studies are needed to clarify the effects of railway and aircraft noise on obesity due to inconsistencies in previous research findings.^31^, ^32^ This ongoing research is crucial for a better understanding of the relationship between noise pollution and obesity, which can have significant implications for public health.

### 3.6 Noise pollution and unhealthy behavior

Recent studies have suggested that exposure to noise pollution might influence unhealthy lifestyle behaviors. Specifically, two studies focusing on road traffic noise and physical activity have revealed associations between noise exposure and reduced physical activity levels.^33^, ^34^ Interestingly, these studies found that noise primarily influenced whether individuals engaged in leisure‐time sports activities at all, rather than the actual amount of time they spent on physical activities each week. Additionally, one study examining the relationship between road traffic noise and lifestyle factors such as smoking and alcohol consumption found positive associations in a cross‐sectional design.^35^ In other words, individuals exposed to higher levels of road traffic noise were more likely to report higher levels of alcohol consumption and smoking. However, these associations did not hold up in longitudinal analyses, which suggests that the relationship between noise exposure and these lifestyle risk factors may be more complex and require further investigation. Despite these findings, it is important to note that the overall quality of evidence linking noise exposure to lifestyle risk factors remains relatively low. While these studies provide intriguing insights, more research is needed to thoroughly examine and validate the hypothesis that transportation noise, through its impact on stress, annoyance, anxiety, and sleep disturbance, contributes to unhealthy lifestyle choices.

### 3.7 Psychiatric disorders and noise pollution

**Figure 2:**
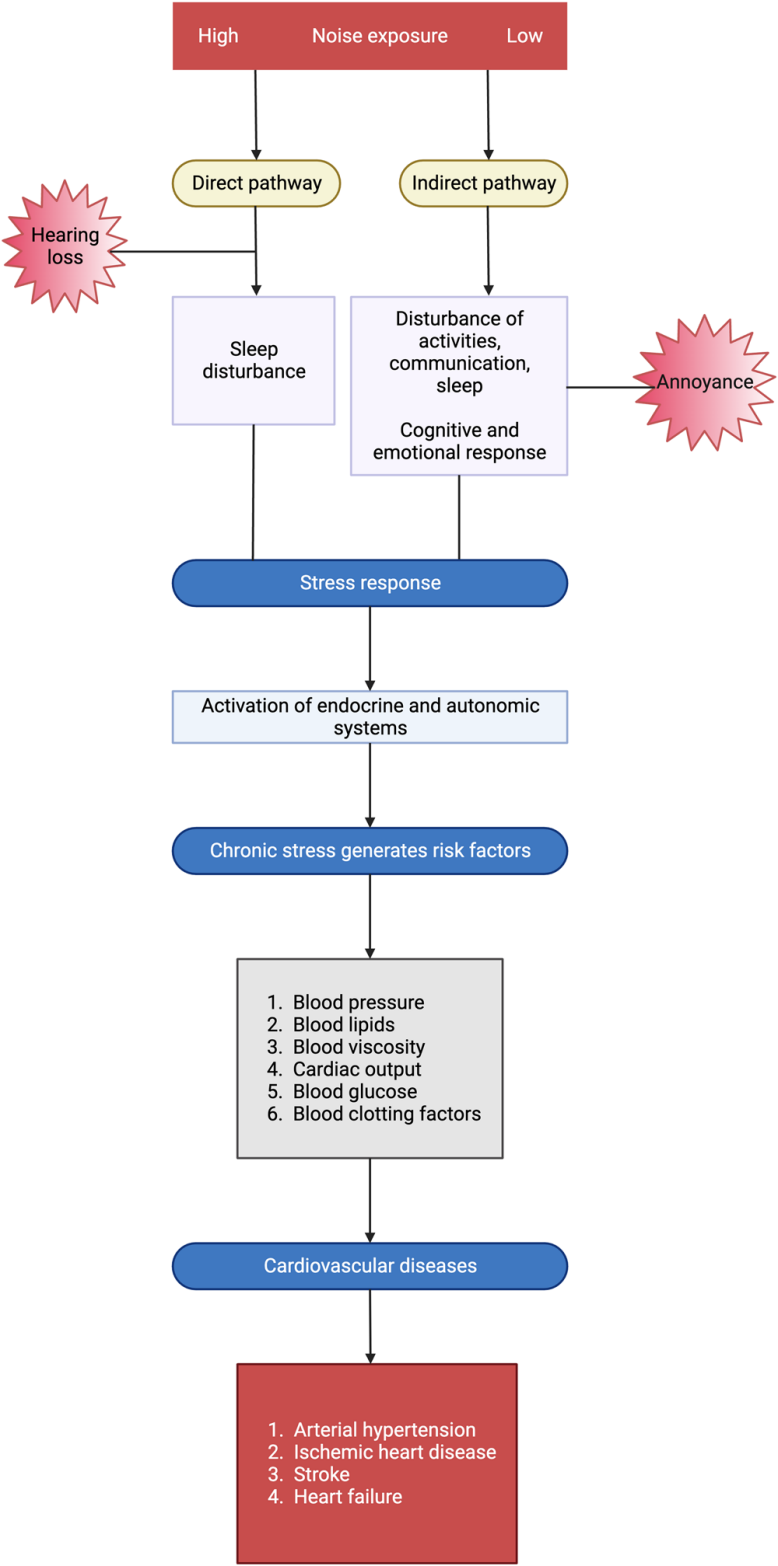
Noise sources and levels.

A potential link between transportation noise and depression has been suggested, but initially, the WHO expert panel concluded that the quality of evidence supporting this connection was rated as “very low.”^36^ However, subsequent research has provided more insights into this relationship. Two longitudinal studies conducted since the WHO evaluation have all indicated an association between road traffic noise and an increased risk of depression.^14^, ^37^ These studies have primarily relied upon such as the use of antidepressant medications and interview‐based assessments of depression symptoms. As a result, the evidence for the association between road traffic noise and depression has been upgraded to “low quality” as of 2020. One challenge in studying the relationship between noise and depression is the wide range of definitions and assessment methods used. Studies have employed various criteria, including self‐reported depression, interviews, the use of antidepressants, and hospital admissions, making it challenging to directly compare findings across different studies.^38^ Therefore, there is a need for more longitudinal research that employs standardized definitions and assessments of depression to provide more clarity on this connection. Importantly, depression itself is considered a cardiovascular risk factor, and individuals with depression are at a higher risk of developing CVD and heart failure.^39^ Additionally, anxiety has also been found to be elevated in individuals exposed to noise, particularly aircraft noise and other forms of environmental noise. Noise pollution triggers an annoyance reaction, reflecting the mental stress experienced by individuals exposed to various noise sources.^40^ Recent evidence suggests that noise can cause sleep disturbances, which, in turn, can lead to cerebral inflammation, oxidative stress, endothelial dysfunction, astrocyte dysfunction, and alterations in the immune system.^41^ These factors have been proposed to contribute to the development of dementia and Alzheimer's disease.^42^ Experimental studies in mice have demonstrated that continuous exposure to aircraft noise induces cerebral oxidative stress, neuroinflammation, endothelial dysfunction, and activation of astrocytes and microglia within just a few days of exposure.^43^ A population‐based study investigated the impact of transportation noise on dementia. It included over 100 000 participants with incident dementia, including Alzheimer's disease, vascular dementia, and Parkinson's disease‐related dementia.^44^ The study found that 10 years of mean exposure to road traffic and railway noise, especially at the highest levels, was associated with an increased risk of all‐cause dementia and specific subtypes, particularly Alzheimer's disease. Importantly, Alzheimer's disease is closely associated with CVD, emphasizing the complex interplay between noise pollution, mental health, and cardiovascular outcomes. Figure 2 shows noise levels and its sources.

## 4 CVDS AND NOISE POLLUTION

### 4.1 Ischemic heart disease

The WHO report and noise guidelines for the European region in 2018, which included studies published up to the end of 2014, identified a significant association between road, aircraft, and railway noise exposure and ischemic heart disease. However, the quality and strength of the evidence supporting these associations were generally rated as low or very low. This assessment was primarily due to limitations in the noise exposure assessment methods used in these studies. Many older studies relied on less precise methods such as postal codes to estimate noise exposure levels, which often provided only an approximation of the noise at the center of an area rather than the actual noise levels experienced at specific addresses.^45^ Additionally, some studies used noise maps without detailed information on true noise levels, potentially leading to underestimations of the health effects associated with noise pollution, particularly in urban areas with small‐scale, heterogeneously distributed road traffic noise. It is also essential to consider factors like whether people sleep with open or closed windows when studying the association between noise and health. Among examined noise, road traffic noise showed the most significant association with the incidence of ischemic heart disease. Specifically, the risk increased by 8% (relative ratio: 1.08; 95% CI: 1.01–1.15) for every 10 dB(A) increase in noise level (*L*_den_).^15^ The quality of evidence from longitudinal studies assessing this association was rated as high. Since then, several studies have reported significant associations between aircraft noise exposure and CVD, including acute mortality. For example, a study in Zurich, Switzerland, found that nighttime deaths from CVD were significantly associated with noise exposure levels in the 2 h preceding death, especially for women and those living in areas with low road and railway background noise.^46^ The study suggested that nighttime aircraft noise could trigger acute cardiovascular mortality, with a population‐attributable fraction estimated at 3%. Another study by Vienneau et al. reported associations between road traffic, railway, and aircraft noise and CVD mortality.^47^ The hazard ratios were 1.029 (95% CI: 1.024–1.034), 1.013 (95% CI: 1.010–1.017), and 1.003 (95% CI: 0.996–1.010) per 10 dB(A) *L*_den_, respectively. These associations were also significant for MI mortality and other CV outcomes, such as blood pressure‐related issues, ischemic heart disease, and stroke. Importantly, the associations were often linear and sometimes observed at noise levels starting below 40 dB(A) *L*_den_ for road traffic and railway noise. Furthermore, the Study on Air Pollution and Lung and Heart Diseases in Adults study identified epigenetic effects associated with transportation noise and air pollution exposure.^48^ Specifically, DNA methylation changes were observed, with distinct and shared enrichments for pathways related to inflammation, cellular development, and immune responses.

### 4.2 Heart failure

The WHO guidelines on noise pollution did not specifically evaluate the impact of noise on heart failure and atrial fibrillation, which are significant cardiovascular outcomes. However, several studies have explored the effects of transportation noise on the risk of heart failure and atrial fibrillation. Regarding heart failure, researchers have conducted five longitudinal studies, including two classical cohorts and three population‐based studies conducted in London, Switzerland, and the Rhine‐Main region.^49^, ^50^, ^51^, ^52^, ^53^ These studies consistently demonstrate an association between exposure to road traffic, railway, and aircraft noise and the incidence and mortality related to heart failure. The observed increase in risk ranges from 2% to 8% per 10 dB(A) increase in noise levels. In contrast, research on the relationship between noise and atrial fibrillation is relatively limited, with only a few studies examining this association. Some of these studies have suggested a positive association between noise exposure and atrial fibrillation, indicating that noise pollution might be a contributing factor to this highly prevalent cardiovascular condition.^49^, ^54^ However, it is worth noting that other studies have reported null results, meaning they did not find a significant association. This variability in findings underscores the need for more research to better understand the potential link between noise pollution and atrial fibrillation.

### 4.3 Cerebrovascular disease

The WHO expert panel assessed the quality of evidence regarding the association between noise pollution and stroke as moderate.^54^ This evaluation was based on a review of five prospective studies: one study focused on stroke incidence and found an increased risk associated with road traffic noise, while the other four studies examined cerebrovascular mortality but did not find a significant association.^49^, ^50^, ^55^ Since that assessment, four additional studies investigating the relationship between transportation noise and incident stroke have been conducted. Two of these studies involved extensive population‐based research covering entire cities or regions (London and Frankfurt) and found that road traffic noise exposure was linked to an increased risk of stroke.^56^, ^57^ However, smaller classical cohort studies from Sweden, Norway, and the United Kingdom, which included fewer cases (ranging from 900 to 1900) but employed a more comprehensive adjustment strategy, did not observe a significant association between noise exposure and stroke risk. Moreover, two large population‐based studies conducted in London and Switzerland suggested that road traffic noise and potentially aircraft noise might be associated with an elevated risk of stroke mortality, particularly ischemic stroke.

## 5 NOISE AND ENDOTHELIAL DYSFUNCTION

**Figure 3:**
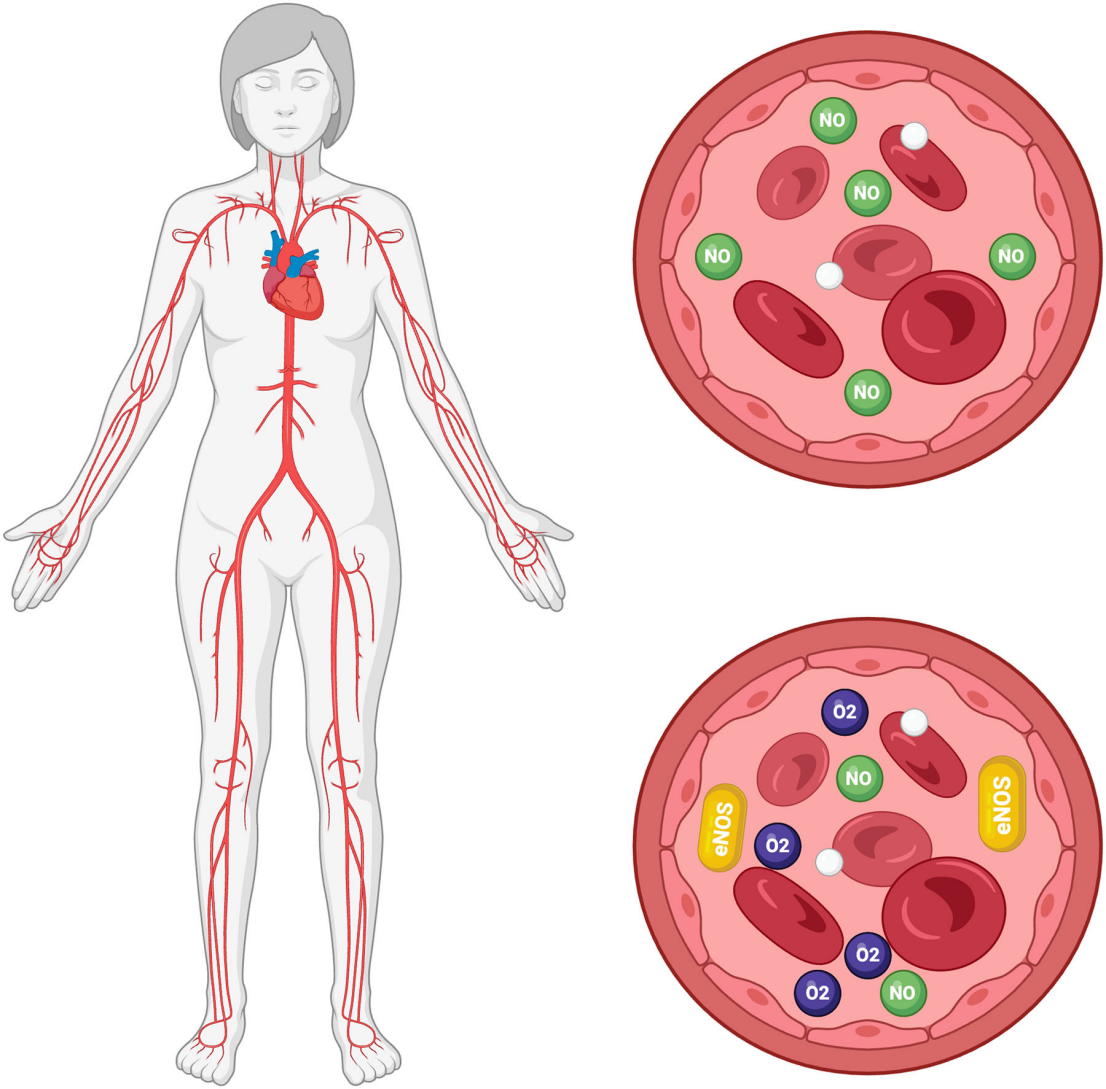
Mechanism of endothelial dysfunction in arteries.

Endothelial function serves as a valuable biomarker for subclinical atherosclerosis and is typically assessed in individuals with cardiovascular risk factors such as high blood pressure, diabetes, elevated cholesterol levels, and chronic smoking, all of which contribute to oxidative stress.^58^ Increased stress hormone levels, which can be triggered by factors like noise pollution, are often accompanied by the production of vascular reactive oxygen species (ROS), indicating oxidative stress, and heightened inflammation marked by increased levels of inflammatory cells like macrophages and cytokines such as interleukin‐6.^8^ Elevated oxidative stress generally leads to endothelial dysfunction due to increased degradation of nitric oxide (NO), a crucial signaling molecule for the endothelium. NO reacts with superoxide, ROS, to produce the highly reactive peroxynitrite, which can inhibit the activity of essential vasodilatory enzymes like prostacyclin synthase and guanylyl cyclase, impairing vasodilation. Additionally, peroxynitrite can directly hinder the activity of endothelial NO synthase (eNOS) by oxidizing its cofactor tetrahydrobiopterin, causing eNOS to shift from producing NO to producing endothelial dysfunction.^59^ Endothelial dysfunction in peripheral arteries often correlates with dysfunction in coronary arteries, emphasizing its role as a systemic predictor of CVD. The processes leading to endothelial dysfunction are complex and involve various mechanisms, including oxidative stress and inflammation.^60^ To investigate whether transportation noise could induce endothelial dysfunction, studies were conducted on the effects of nighttime aircraft noise exposure on healthy volunteers. During noise exposure nights, recorded aircraft noise was played back to the subjects. These studies revealed that nighttime aircraft noise exposure led to reduced sleep quality, increased stress hormone levels, endothelial dysfunction, and decreased pulse transit time (indicating sympathetic nervous system activation) in healthy individuals. In subjects with established coronary artery disease, the adverse effects of nighttime aircraft noise on endothelial dysfunction were even more pronounced.^61^ Similar endothelial dysfunction was observed in response to nighttime railway noise. Importantly, these noise‐induced endothelial dysfunctions improved when antioxidant vitamin C was administered, suggesting that oxidative stress plays a significant role in these effects. It is worth noting that mechanistic studies on the effects of noise on vascular function have been relatively rare, and in some cases, white noise has been used as a noise source at high decibel levels, around 100 dB(A), which is considerably louder than typical environmental noise.^62^ Studies involving animals exposed to white noise at such high levels showed impaired endothelium‐dependent vasodilation, increased sensitivity to vasoconstrictors, and hypertension.^63^ These findings are qualitatively similar to studies using aircraft noise, but it is important to highlight that white noise is not representative of typical environmental noise and can even improve sleep in individuals dealing with high levels of noise pollution. Figure 3 illustrates the mechanism of endothelial dysfunction in arteries.

## 6 MYOCARDIAL FUNCTION AND NIGHT‐TIME NOISE

To investigate the impact of noise event frequency on vascular and myocardial function, a prospective randomized trial involved 70 individuals with established CVD or increased cardiovascular risk.^64^ In this study, two different aircraft noise scenarios (60 noise events [Noise60] and 120 noise events [Noise120]) and one control scenario were examined over three study nights. The noise scenarios featured either Noise60 or Noise120 in one night, both with comparable equivalent noise levels (Leq), averaging around 45 dB(A).^64^ In contrast, the control night had a mean noise level of 37 dB(A). The findings revealed that during the control night, participants had a flow‐mediated dilation (FMD) of 10.02 ± 3.75%, indicating relatively healthy vascular function. However, after exposure to both noise scenarios, FMD decreased, measuring 7.27 ± 3.21% for Noise60 and 7.21 ± 3.58% for Noise120.^64^ This indicates a significant impairment of vascular function in response to aircraft noise, with similar Leq but different numbers of noise events. Furthermore, the study assessed sleep quality and found that it was compromised after noise exposure in both noise scenarios. The research also included serial echocardiographic assessments, which showed an increase in the ratio of peak E‐ and E′‐wave velocity (E/E′ ratio), an indicator of diastolic function. During the control E/Eht, the E/E′ ratio was 6.83 ± 2.26, while it increased to 7.21 ± 2.33 for Noise60 and 7.83 ± 3.07 for Noise120 within the three exposure nights.^64^ This suggests that nighttime aircraft noise exposure has adverse effects on myocardial function, specifically diastolic dysfunction, which appeared to be more pronounced with a higher number of noise events.

## 7 NOISE AND AIR POLLUTION CO‐EXPOSURE

Comparative studies assessing the burden of disease have revealed some alarming insights into the adverse health effects of environmental factors. Air pollution stands out as the leading environmental cause of disability‐adjusted life years lost (DALYs). However, environmental noise is ranked as the second major contributor to DALYs in Europe, surpassing the impact of certain well‐known pollutants like lead, ozone, and dioxins.^65^ It is essential to note that air pollution and noise often share common sources, such as heavy industry, aircraft, railways, and road vehicles. Consequently, people often face coexposure to both air pollution and noise, compounding their health risks. Research estimates indicate that the social cost of noise and air pollution, which includes excess deaths and diseases, could reach nearly one trillion euros in the European Union. To put this into perspective, the social cost of alcohol in the EU is estimated at 50–120 billion euros, and smoking‐related costs are estimated at a staggering 544 billion euros.^66^ Some studies have recognized the interplay between air pollution and noise, making adjustments for these factors in their analyses. For instance, in the European Study of Cohorts for Air Pollution Effects, researchers found a positive association between self‐reported hypertension and particulate matter with a diameter of 2.5 μm.^67^ Interestingly, when they adjusted for road noise, the estimate of this association decreased. Similarly, the Nord‐Trøndelag Health Study established a significant link between long‐term exposure to road traffic noise and ambient air pollution with CVD biochemical parameters. In recent research, scientists have examined the potential synergistic effects of particulate matter and noise exposure.^68^ They conducted experiments involving mice exposed to ambient particulate matter and noise for 3 days, specifically aircraft landing and take‐off events. The results were concerning, as both stressors independently led to endothelial dysfunction, increased blood pressure, oxidative stress, and inflammation. When combined, they produced an additive negative impact on endothelial function, particularly pronounced in cerebral and retinal arterioles. Notably, the research indicated that noise predominantly affects the brain, while particulate matter affects the lungs. This combination of stressors results in heightened oxidative stress and inflammation, potentially through a mechanism involving systemic inflammation induced by particulate matter and stress hormone signaling triggered by noise exposure.^69^ Furthermore, the studies revealed an additive upregulation of angiotensin‐converting enzyme 2 in the lung, which might provide insight into the increased vulnerability to COVID‐19 infection reported in populations in high air‐ and noise‐polluted areas.

## 8 STRATEGIES TO MITIGATE NOISE POLLUTION

Addressing noise pollution requires a nuanced approach because various noise sources necessitate tailored mitigation strategies. Broadly, noise abatement measures can be categorized as active or passive.^70^ Passive measures focus on structural modifications to reduce noise, such as installing soundproof windows or insulating roofs and roller blind boxes. On the other hand, active noise abatement measures aim to reduce noise at its source. For example, in the case of aircraft noise, these measures could involve changes in flight patterns, such as continuous descending approaches, flying at higher altitudes, steeper landings, or using Global Positioning System‐guided approaches to avoid flying over residential areas. Additionally, newer engine technologies can contribute to noise reduction. Night flight bans have proven to be highly effective in mitigating noise pollution from aircraft. Railway noise can be effectively reduced through active measures like speed reductions, noise barriers, and the use of last‐generation brake blocks, resulting in noise reductions of 10–20 dB(A).^70^ Similarly, road noise can be actively managed through strategies like using quieter road surfaces, low‐noise tires, promoting eco‐friendly driving practices, and the adoption of electric cars. Passive measures for railway and road noise include improving building insulation and designing bedrooms along the least exposed façade to minimize noise intrusion. It is worth noting that electric cars, while beneficial for reducing air pollution, may have a limited impact on reducing road traffic noise, particularly at speeds above 30–35 km/h, as road noise is primarily generated by the interaction between tires and road surfaces. In this context, the transition from combustion engine cars to electric vehicles may result in a minimal noise reduction of approximately 1 dB(A). When considering the costs and benefits of noise abatement measures for road and rail traffic, studies show that the benefits consistently outweigh the costs.^70^ A combination of strategies is often the most cost‐effective approach to mitigate noise pollution effectively. Furthermore, successful mitigation strategies for environmental stressors may extend to urban planning. Increasing green spaces in cities can have multifaceted benefits.^71^ More greenery helps counteract the adverse effects of urban heat islands, which occur due to extensive concrete surfaces. These benefits include reduced hospitalizations, lower rates of CVD, enhanced biodiversity, improved sleep quality, and even a healthier indoor microbiome. Research has shown that environmental risk factors, including air pollutants, noise pollution, and the lack of green space, are independently associated with a higher risk of T2DM.^19^ When these risk factors are considered together, they lead to a cumulative greater risk of T2DM. Access to more green spaces appears to be a crucial factor in mitigating these health risks. Moreover, green spaces have the potential to enhance physical, mental, and community health, underscoring their importance in urban planning and environmental policy.

## 9 CONCLUSION

In conclusion, noise pollution is a pervasive and often underestimated environmental health concern with far‐reaching implications for public well‐being. This article has explored the various dimensions of noise pollution, its sources, and the extensive evidence linking it to adverse health effects, including cardiovascular problems, sleep disturbances, and psychological stress. Moreover, the significant economic burden associated with noise‐related health issues emphasizes the urgency of addressing this issue. While the consequences of noise pollution are well‐documented, there is a growing recognition of the need for comprehensive strategies to mitigate its impact.

## CONFLICT OF INTEREST STATEMENT

The authors declare no conflict of interest.

## Data Availability

Data sharing is not applicable to this article as no new data were created or analyzed in this study.
